# Ezrin interacts with S100A4 via both its N- and C-terminal domains

**DOI:** 10.1371/journal.pone.0177489

**Published:** 2017-05-11

**Authors:** Beáta Biri-Kovács, Bence Kiss, Henrietta Vadászi, Gergő Gógl, Gyula Pálfy, György Török, László Homolya, Andrea Bodor, László Nyitray

**Affiliations:** 1 Department of Biochemistry, Eötvös Loránd University, Budapest, Hungary; 2 Institute of Chemistry, Laboratory of Structural Chemistry and Biology, Eötvös Loránd University, Budapest, Hungary; 3 Molecular Cell Biology Research Group, Institute of Enzymology, Research Centre for Natural Sciences, Budapest, Hungary; Russian Academy of Medical Sciences, RUSSIAN FEDERATION

## Abstract

Ezrin belongs to the ERM (ezrin, radixin, moesin) protein family that has a role in cell morphology changes, adhesion and migration as an organizer of the cortical cytoskeleton by linking actin filaments to the apical membrane of epithelial cells. It is highly expressed in a variety of human cancers and promotes metastasis. Members of the Ca^2+^-binding EF-hand containing S100 proteins have similar pathological properties; they are overexpressed in cancer cells and involved in metastatic processes. In this study, using tryptophan fluorescence and stopped-flow kinetics, we show that S100A4 binds to the N-terminal ERM domain (N-ERMAD) of ezrin with a micromolar affinity. The binding involves the F2 lobe of the N-ERMAD and follows an induced fit kinetic mechanism. Interestingly, S100A4 binds also to the unstructured C-terminal actin binding domain (C-ERMAD) with similar affinity. Using NMR spectroscopy, we characterized the complex of S100A4 with the C-ERMAD and demonstrate that no ternary complex is simultaneously formed with the two ezrin domains. Furthermore, we show that S100A4 co-localizes with ezrin in HEK-293T cells. However, S100A4 very weakly binds to full-length ezrin *in vitro* indicating that the interaction of S100A4 with ezrin requires other regulatory events such as protein phosphorylation and/or membrane binding, shifting the conformational equilibrium of ezrin towards the open state. As both proteins play an important role in promoting metastasis, the characterization of their interaction could shed more light on the molecular events contributing to this pathological process.

## Introduction

**Fig 1:**
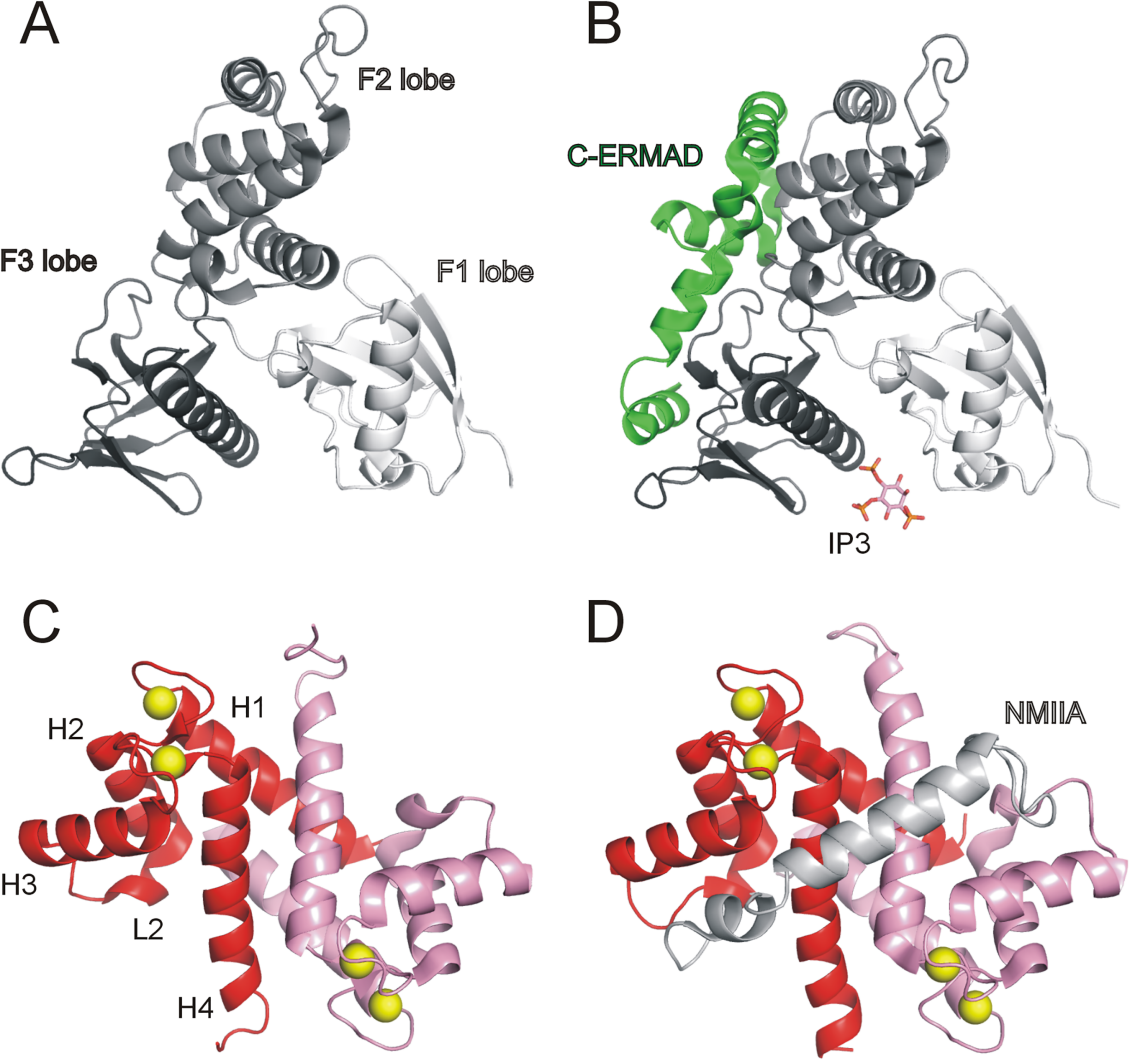
Structural overview of ezrin and S100A4 proteins. (A) Crystal structure of the human ezrin N-ERMAD (PDB ID: 4RMA). (B) Crystal structure of full-length human ezrin (PDB ID: 4RM8). Note that the 160 residue-long α-helical domain, which connects the N-ERMAD and the C-ERMAD, and the N-terminal 39 residues of the C-ERMAD (Val477-Glu515) are not visible in the crystal structure. Crystal structure of mouse radixin N-ERMAD in complex with inositol-(1,4,5)-trisphosphate (IP3; PDB ID: 1GC6) was used to demonstrate the putative lipid-binding site of ezrin. (C) Crystal structure of calcium-bound S100A4 (PDB ID: 3C1V) and (D) calcium-bound S100A4 complexed with non-muscle myosin IIA (NMIIA) C-terminal fragment (PDB ID: 3ZWH).

Ezrin is a member of the ERM protein family and is responsible for linking the plasma membrane and the cytoskeleton; therefore it has important roles in cell adhesion, migration and cell growth [1]. It also participates in pathological processes such as cancer cell invasion and metastasis [2]. Ezrin consists of three domains: the FERM domain that is located at the N-terminal region (called N-ERMAD, ~300 amino acids), an α-helical linker region (~160 residues) and a C-terminal domain (called C-ERMAD, ~ 200 amino acids). N-ERMAD has a cloverleaf-like structure with three lobes (F1, F2 and F3) (Fig 1A), and has a role in binding to plasma membrane-bound proteins such as syndecan-2, CD44 and a set of adhesion molecules [3–5]. Transmembrane proteins usually bind to the third lobe of the N-ERMAD [6], while the C-ERMAD is responsible for binding to actin filaments [7]. In a dormant state, the C-terminal region binds to the second and third lobe of the N-terminal domain (Fig 1B), masking the plasma membrane- and actin-binding sites [8]. Ezrin can be activated by binding to phosphatidylinosytol-(4,5)-bisphosphate (PIP_2_) through the F1 and F3 lobes of the N-ERMAD [9, 10] and by phosphorylation of Thr567 in the C-ERMAD by numerous AGC-type kinases (i.e. ROCK [11], PKCα [12], Akt2 [13] or GRK2 [14]). Upon activation, the self-associated structure opens up and the released C-ERMAD will be able to bind to various partners, such as F-actin and exert its function [15]. Several high-throughput studies indicated other important phosphorylation sites (on specific tyrosine residues), however they have not yet been investigated in detail [16]. These results suggest that activation of ezrin could be a more complex process that is generally oversimplified in the literature.

S100 proteins are vertebrate-specific members of the EF-hand containing Ca^2+^-binding superfamily with more than 20 paralogs in the human proteome. They are small, mostly homodimeric proteins, where each monomer can bind two calcium ions. Upon Ca^2+^-activation, a large conformational switch occurs, exposing a shallow hydrophobic groove in each subunit to allow binding of S100 proteins to various intra- and extracellular partners (Fig 1C) [17]. Recently, it was revealed that S100 proteins can also bind their protein partners in an asymmetrical manner, where both canonical binding grooves are occupied by one chain of the interacting partner (Fig 1D) [18, 19]. S100A4 (metastasin), a member of this family, has multiple known binding partners such as non-muscle myosin IIA (NMIIA), annexin A2 and p53 [19–21]. By disrupting NMIIA filaments, S100A4 has a role in cytoskeleton-linked processes, such as cell adhesion, migration and invasion. It is overexpressed in several metastatic tumors, therefore it is a possible therapeutic target in cancer research [22].

It was previously demonstrated that S100P (but not the two other tested S100 proteins: S100A1 or S100A11) binds to the second lobe (F2) of the N-ERMAD [23, 24]. This binding event promotes ezrin and F-actin interaction through ezrin activation, leading to enhanced transendothelial migration of tumor cells [24]. However, it was also shown that S100P and PIP_2_ compete for ezrin binding suggesting that the mechanism of S100P-mediated enhancement of ezrin-related functions must be more complicated than a simple conformational activation process. More recently it was demonstrated that the S100A8/A9 heterodimer in complex with inducible NO synthase (iNOS) directs the selective S-nitrosylation of the ERM proteins ezrin and moesin [25].

It is well established that the interactomes of the closely related S100 paralogs partially overlap [26–29], therefore, we investigated whether other S100 family members bind to ezrin. Using tryptophan fluorescence, stopped-flow kinetics, fluorescence polarization, CD and NMR spectroscopy methods we characterized the interaction between ezrin domains and S100A4. We show here that the N-ERMAD mediates specific and similar affinity interaction with S100A4 as with S100P. Interestingly, S100A4 (unlike other S100 proteins) binds to the C-terminal domain of ezrin (C-ERMAD). Although we could only detect a very low affinity between S100A4 and full-length ezrin *in vitro*, the interaction was validated in living cells by FRET and also by colocalization study, indicating that special cellular conditions, which promote ezrin conformational activation, are required for the complex formation. Based on our results, a possible mechanism for S100A4-mediated regulation of ezrin function is proposed.

## Materials and methods

### Production of recombinant constructs and proteins

S100 proteins and variants were cloned, expressed and purified as previously described [18, 30]. Briefly, they were cloned into a modified pET expression vector with N-terminal TEV cleavable hexahistidine-tag (His_6_-tag). S100A4 mutants were generated by the”megaprimer” method [31]. They were expressed in *E*. *coli* BL21(DE3) cells and purified on a Ni^2+^-affinity column (Bio-Rad) followed by the cleavage of the His_6_-tag with TEV protease. After complete cleavage, samples were supplemented with Ca^2+^ and were injected into a phenyl-Sepharose column. Elution was done by using EDTA-containing buffer; then the eluted fractions were concentrated and stored at −70°C. ^15^N-labeled S100A4-Δ9 was expressed and purified as described in [32].

The cDNA encoding human ezrin (Uniprot code: P15311) was produced from mRNA derived from A431 epithelial carcinoma cells. The amplified gene was then cloned into the same pET-based expression vector containing N-terminal His_6_-tag and a TEV protease cleavage site. The N-ERMAD (Met1-Lys296) and its F2 lobe (Glu87-Glu199) were cloned similarly. Thr567Asp mutation was introduced by the “megaprimer” method. Proteins were expressed in *E*. *coli* Rosetta(DE3) cells (Novagen) using standard techniques. Protein purification was performed on Ni^2+^-affinity columns in 50 mM NaH_2_PO_4_ pH 8 and 300 mM NaCl. His_6_-tag was removed by TEV protease cleavage, while the sample was dialyzed against a buffer containing 50 mM Tris pH 7, 100 mM NaCl. High purity was achieved by cation exchange chromatography (HiTrap SP HP column, GE Healthcare Life Sciences) or via size exclusion chromatography using an in-house packed 10/300 Superdex 75 column in case of the F2 lobe. Pure fractions were collected and concentrated to 200–500 μM with Amicon Ultra centrifugation filter units (Millipore) and final aliquots were stored at −70°C. The C-ERMAD (Cys-Lys516-Leu586) and the C-ERMAD^516–560^ (Cys-Lys516-Leu560) were cloned with an N-terminal TEV cleavable GST fusion tag. Cysteine residue was cloned for subsequent fluorescent labeling. GST-fusion peptides were purified using glutathione-Sepharose 4B resin (GE Healthcare). After TEV cleavage, GST and TEV were precipitated by heat denaturation. The protein precipitation was centrifuged and the supernatant was further purified by reverse-phase HPLC on a Jupiter 300 C5 column (Phenomenex). The purity of recombinant ezrin variants and S100 proteins was verified by Tris-Tricine SDS-PAGE (S1 Fig).

The fluorescein-labeled NMIIA peptide (Fl-NMIIA, Asp1908-Ala1935) was synthesized in-house by solid-phase techniques using an ABI 431A peptide synthesizer (Applied Biosystems) and standard N-(9-fluorenyl)-methoxycarbonyl chemistry, labeled at the N-terminus with 5-carboxyfluorescein and separated by reverse phase HPLC.

For FRET and colocalization experiments, coding sequences of wild-type S100A4 and its C-terminally truncated Cys81Ser mutant (S100A4-SerΔ13) were cloned into pmCherry-C1 eukaryotic expression vector (using restriction sites *Kpn*I-*Bam*HI), while ezrin was cloned into pEGFP-C1 plasmid (using restriction sites *Xho*I-*Sac*II). The positive control (pEGFP-mCherry, 100% FRET) was cloned using *Xho*I-*Eco*RI restriction sites, where GFP was connected with a 7 amino acid linker (SGLRSRA) to mCherry.

### Steady-state tryptophan fluorescence measurements

2 μM full-length ezrin, its phosphomimicking mutant ezrin^T567D^, N-ERMAD and F2 lobe were titrated with S100 proteins in a buffer containing 20 mM HEPES pH 7.5, 150 mM NaCl, 0.5 mM TCEP and 1 mM CaCl_2_ (or 0.1 mM EDTA where indicated) for 10 min at 25°C. Fluorescence was measured in 384-well plates (Corning #3676) using Synergy H4 multi-mode microplate reader (BioTek) setting excitation and emission wavelengths to 295 ± 2.5 nm and 340 ± 2.5 nm, respectively. *K*_d_ values were calculated by fitting the data to a quadratic binding equation using software Origin Pro 8 (OriginLab Corp.).

### Tryptophan fluorescence based kinetic measurements

Fast kinetic measurements were performed with the stopped-flow instrument SFM-300 (Bio-Logic) with excitation at 297 nm. Fluorescence emission from tryptophan residues was observed through a 340 nm band-pass filter (Comar Optics). All reactions were measured at 25°C in a buffer containing 20 mM HEPES pH 7.5, 150 mM NaCl, 0.5 mM TCEP and 1 mM CaCl_2_. Post-mixing N-ERMAD and F2 lobe concentrations were fixed to 1 μM. Transients were fitted using a single exponential function. Amplitude versus concentration plots were fitted by a quadratic binding equation, while in the case of the observed rate constant versus concentration plots a hyperbola was fitted to the data points using Origin Pro 8 (OriginLab Corp.).

### Fluorescent labeling

The C-ERMAD fragments were labeled selectively at the N-terminal cysteine with a 2-fold excess of Alexa Fluor 568 C_5_ maleimide (Molecular Probes) in 100 mM HEPES pH 7.0 by incubating the samples for 3 hours in the dark at room temperature. The fluorescein-conjugated peptides (denoted as Fl-C-ERMAD) were separated from both the non-reacted 5-IAF and the unconjugated peptide by RP-HPLC.

### Fluorescence polarization assay

Fl-C-ERMAD or Fl-C-ERMAD^516–560^ (50 nM) was titrated with S100 proteins in a buffer consisting of 20 mM HEPES pH 7.5, 150 mM NaCl, 0.1 mM TCEP, 1 mM CaCl_2_ (or 0.1 mM EDTA) and 0.05% Tween-20. Fluorescence polarization was measured in 384-well plates (Corning #3676) using Synergy H4 multi-mode microplate reader (BioTek). Titration experiments were carried out in triplicates and the average FP signal was used for fitting the data to the quadrative binding equation. In the competitive assays 50 nM Fl-NMIIA complexed with 4 μM S100A4 dimer was titrated with the different ezrin constructs. Titration experiments were carried out in triplicates and the average FP signal was used for fitting the data to a competition binding equation.

### Circular dichroism

CD measurements were performed on a Jasco J-810 spectropolarimeter (JASCO Corporation) using a 0.01 cm path length quartz cuvette. Far-UV spectra were taken in the wavelength range of 200–250 nm in a buffer containing 10 mM Tris pH 7.5, 150 mM NaCl, 0.1 mM TCEP and 1 mM CaCl_2_. The CD spectrum of the N-ERMAD-bound C-ERMAD was calculated by the subtraction of the CD spectrum of the free N-ERMAD from that of the complex presuming that the secondary structure of the N-ERMAD does not change upon complex formation (Fig 1A and 1B) [33]. Similarly, the CD spectra of the S100A4-bound ezrin fragments were calculated by the subtraction of the CD spectrum of free S100A4 from that of the complex presuming that the secondary structure of S100A4 does not change upon complex formation (Fig 1C and 1D) [18, 34]. The CD spectra of the bound and non-bound ezrin fragments were deconvoluted using BeStSel software [35].

### NMR measurements

Measurements were performed on a Bruker Avance III 700 MHz spectrometer equipped with a z-gradient 5-mm probe-head operating at 700.17 MHz for ^1^H and 70.95 MHz for ^15^N nuclei. Typical composition of the NMR samples were: 0.2–0.8 mM ^15^N-labeled S100A4-Δ9, 20 mM MES pH 6.0, 150 mM NaCl, 10 mM CaCl_2_, 5 mM TCEP, 3 mM NaN_3_ and 10% D_2_O. All chemical shifts were referenced to the internal DSS resonance, while ^15^N chemical shift values were referenced indirectly using the corresponding gyromagnetic ratios according to IUPAC convention. Sequence specific assignment of H^N^, N and side chain proton resonances was done using a ^15^N-labeled protein at 300 K and on the basis of standard 3D HSQC-TOCSY (mixing time 70 ms) and 3D HSQC-NOESY (mixing time: 150 ms) measurements and also relying on earlier results from the assigned S100A4-Δ13 protein (BMRB entry code: 25136) [32]. All spectra were processed with TOPSPIN and analyzed using CARA (ETH Zürich) and SPARKY softwares [36, 37].

Interaction studies were performed by addition of unlabeled N-ERMAD and the C-ERMAD. Δ*δ* chemical shift mapping values were calculated as described earlier [32]. In the NMR titration experiment, unlabeled C-ERMAD (7.5 mM) was added gradually to the NMR tube containing ^15^N-labeled S100A4-Δ9 dimer (250 μM). To determine the binding affinity and stoichiometry, ten peaks were selected (Ala2, Leu5, Leu9, Phe27, Asn30, Thr39, Asp67, Asn68, Asp71, Phe72) that were shifted upon the addition of C-ERMAD, due to fast exchange between the free and bound forms. The mean ± SEM of the normalized Δ*δ* values were plotted against C-ERMAD / S100A4-Δ9 dimer molar ratio and the data were fitted to a quadratic binding equation.

Translational diffusion measurements were performed using the *stebpgp1s19* pulse sequence. The strength of the diffusion gradient was varied linearly in 32 steps between 6% and 98% of its maximum value. The applied maximum gradient strength was 45.4 G/cm. Diffusion coefficients were obtained by fitting the decay of the proton signals in the aliphatic region (integrals over chosen regions) according to the Stejskal-Tanner equation using the T1/T2 package of the Bruker TOPSPIN program. The assigned chemical shifts of S100A4-Δ9 and S100A4-Δ9 in complex with the C-ERMAD were deposited in the Biological Magnetic Resonance Bank data base with the following entries: 26946 and 26956, respectively.

### Cell culturing and transient transfection

The HEK-293T human embryonic kidney cell line and the A431 epithelial carcinoma cell line were provided by Drs. Attila Reményi and László Buday, respectively. Cells were maintained in Dulbecco’s Modified Eagle Medium (DMEM, Lonza), supplemented with 10% Fetal Bovine Serum (BioWest) and Penicillin-Streptomycin-Amphotericin B (Lonza). Transient transfection was done using FuGene HD (Promega) transfection reagent according to the manufacturer’s instructions.

### FRET measurements

HEK-293T cells were seeded to μ-Slide (chambered coverslip, 8 wells, Ibidi) and transfected 48 h prior to FRET measurements with the appropriate plasmids. Donor photobleaching was performed on living cells (for 120 s) using a Zeiss LSM710 confocal microscope. Briefly, we compared the bleaching constant (*t*_1/2_) of cells expressing only the donor fluorophore-conjugated protein (pEGFP-C1-ezrin) with the bleaching constants of co-transfected cells (pEGFP-C1-ezrin and pmCherry-C1-S100A4/S100A4-SerΔ13). Transfection with plasmid encoding EGFP conjugated to mCherry (pEGFP-mCherry, 100% FRET) served as a positive control. During measurements, cell medium was changed to CO_2_-independent medium (Thermo Fisher Scientific). Bleaching constants were calculated by ImageJ (Time Series Analyzer V3 plugin) and Origin Pro8 (OriginLab Corp.) software using an exponential decay equation. For every set of sample, at least 80 regions of interest (ROI) were analyzed. For statistical analysis, the Games-Howell test was performed using R 3.2.0 software. The Games-Howell test is based on the Tukey-Kramer test and is an adequate method for pairwise comparisons in case of unequal sample size and inhomogeneous variance [38]. Group variances were tested by the Levene’s method. Differences were considered to be significant if p < 0.05.

### Colocalization studies

A431 epithelial carcinoma cells were seeded to coverglass-containing 24-well plates. After 24 h, cells were transfected with plasmids pmCherry-S100A4 or pmCherry-S100A4-SerΔ13 using FuGene HD transfection reagent. The day after cells were serum-starved for 14 h, followed by EGF stimulation (10 μg/ml, for 15 min, Sigma-Aldrich) and fixed with 4% paraformaldehyde. After permeabilization, immunostaining was performed using monoclonal anti-ezrin antibody (Santa Cruz Biotechnology and Alexa-488 conjugated secondary antibody (Thermo Fisher Scientific). Nuclei were stained by DAPI. Images were taken by Zeiss AxioImager Z1 microscope.

## Results

### S100A4 binds to the N-ERMAD domain of ezrin

**Fig 2:**
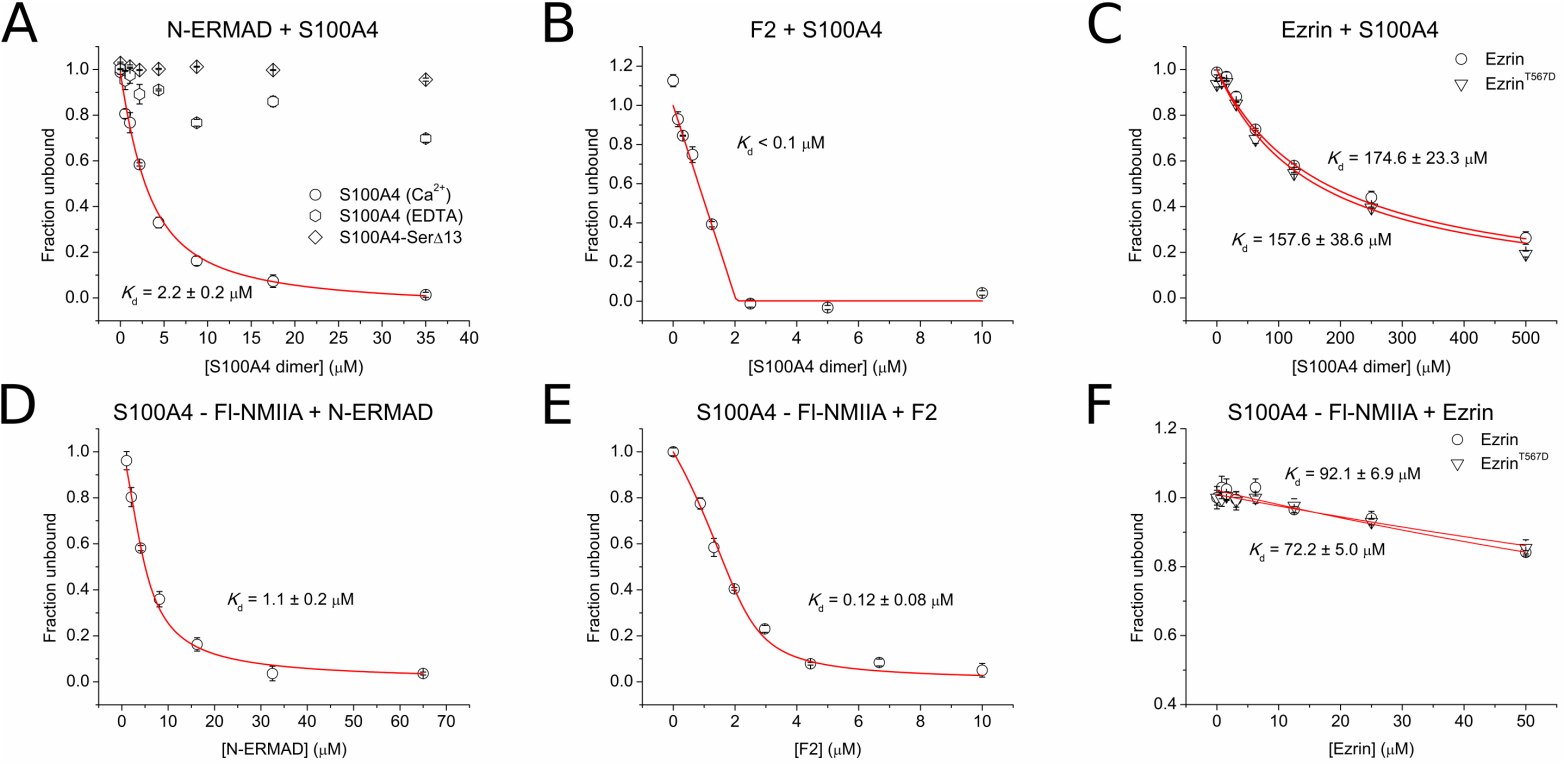
Interaction of the N-ERMAD with S100 proteins. (A-C) Tryptophan fluorescence-based binding experiments were performed using 2 μM N-ERMAD, F2 lobe and ezrin (or ezrin^T567D^), respectively, with S100A4. (D-F) In a competitive FP assay, the known S100A4-partner NMIIA^1908-1937^ peptide (fluorescein-conjugated, 50 nM) was preincubated with 4 μM S100A4 (dimeric concentration) and titrated with the N-ERMAD, F2 lobe or full-length ezrin (or its variant), respectively. Each data point represents the mean ± SEM of three independent experiments. The data were fitted using a quadratic (A-C) or competitive binding equation (D-F) (red line).

Koltzscher and co-workers showed that the N-ERMAD interacts with S100P selectively, i.e. no binding to other S100 proteins could be detected (S100A1 and S100A11 were analyzed) [23]. Recent results demonstrated that S100P, like S100A4, binds to non-muscle myosin isoforms [28] and this finding triggered the study of other ezrin-S100 protein interactions involving S100 paralogs A2, A4, A6 and B in the binding assays. As each of the three N-ERMAD lobes (F1, F2 and F3) contains two tryptophan residues and no tryptophan residue is found in any of the investigated S100 paralogs, S100 binding to the N-ERMAD can be followed by the change in Trp fluorescence intensity. A significant drop in Trp fluorescence intensity was detected upon the titration of the N-ERMAD with S100A4 in the presence of Ca^2+^ (Fig 2A). Trp fluorescence intensity of the N-ERMAD was also changed by binding of other S100 proteins, though only S100P has a binding affinity in the same order of magnitude as S100A4 (S2A Fig). S100A4 and S100P interact with the N-ERMAD with micromolar dissociation constants (2.2 ± 0.2 μM and 1.7 ± 0.3 μM, respectively), while S100A2, S100A6 and S100B bind with low affinity (*K*_d_ ≈ 10–20 μM). Note that S100A4 binding is strictly Ca^2+^-dependent (Fig 2A). To validate the binding experiments, the assay was repeated with an S100A4 deletion mutant lacking the last 13 amino acids and containing Ser instead of Cys at position 81 (S100A4-SerΔ13). The mutation of Cys81 is known to disrupt NMIIA binding [18, 39], furthermore, hydrophobic residues in the C-terminal tail of S100P were shown to be critical for the N-ERMAD binding [24]. According to our expectations, we found that S100A4-SerΔ13 did not bind the N-ERMAD (Fig 2A), therefore we used this mutant as a negative control in the subsequent cellular experiments.

Further characterization of the N-ERMAD-S100A4 interaction was performed using the isolated F2 subdomain. At an F2 concentration of 2 μM only the upper limit of the *K*_d_ could be determined (0.1 μM). As a consequence of the relatively tight binding of S100A4 to the F2 lobe, the stoichiometry of the interaction can be determined and was found to be one S100A4 dimer to an F2 lobe (Fig 2B). Finally, we also investigated the binding of S100A4 to the full-length ezrin. The results show that interaction with the full-length protein under *in vitro* conditions is very weak (174.6 ± 23.3 μM). As phosphorylation of threonine at position 567 contributes to ezrin activation, we also carried out the binding assay using a phosphomimetic ezrin variant ezrin^T567D^. Interestingly, S100A4 showed a similar binding to this mutant also (*K*_d_ = 157.6 ± 38.6 μM) (Fig 2C).

Assuming that both the N-ERMAD and the F2 subdomain would compete with the well-characterized S100A4 interacting protein NMIIA for binding to S100A4, competitive fluorescent polarization (FP) was used as a parallel technique to determine the equilibrium binding constants of ezrin-S100A4 interactions. A fluorescein-labeled NMIIA (Fl-NMIIA) peptide was used as a tracer that binds to S100A4 with micromolar affinity [18, 19] (S2B Fig). When Fl-NMIIA was complexed with S100A4 and titrated with either the N-ERMAD or the F2 lobe, a decrease in the FP signal was detected showing that both ezrin constructs compete with the NMIIA peptide for S100A4 binding (Fig 2D and 2E). Fitting the data to a competitive binding equation resulted in the dissociation constants of 1.07 ± 0.19 μM and 0.12 ± 0.08 μM for the N-ERMAD-S100A4 and the F2-S100A4 interaction, respectively. Note that these values are in good agreement with those determined by the tryptophan fluorescence-based measurements. When the full-length ezrin and its mutant ezrin^T567D^ were used as a competitor, only a very low level of competition was detectable in these experiments (*K*_d_ = 92.1 ± 6.9 and 72.2 ± 5.0 μM, respectively) reinforcing our conclusion that ezrin predominantly resides in a closed, dormant state, which is incapable of interacting with S100A4 (Fig 2F).

### Fast-kinetic analysis of the N-ERMAD-S100A4 interaction

**Fig 3:**
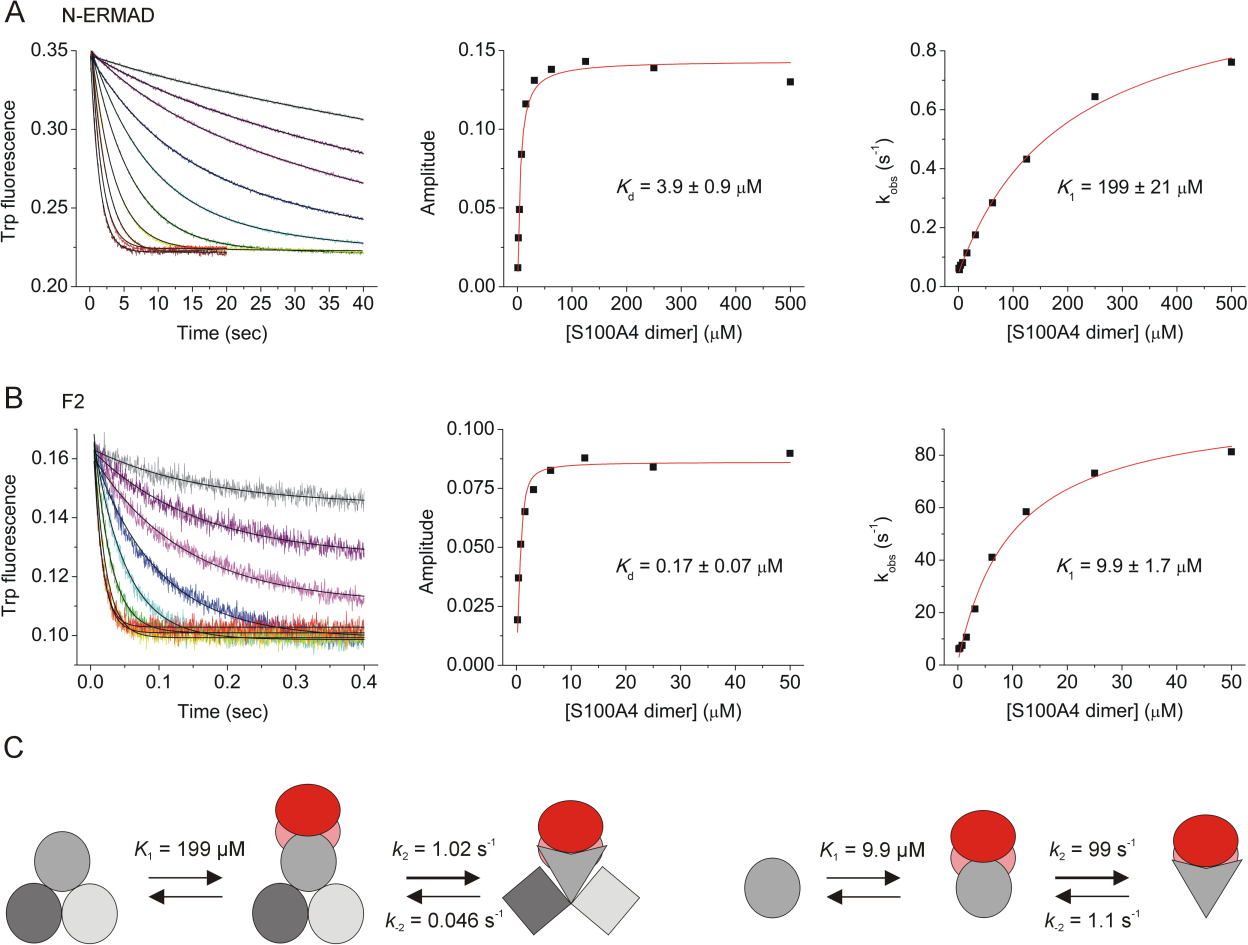
Transient kinetic analysis of the S100A4-N-ERMAD interaction. (A, B) 2 μM N-ERMAD or F2 lobe (respectively) was mixed with an equal volume of S100A4 in different concentrations and a decrease in intrinsic Trp fluorescence was monitored over time (left panel). The equilibrium dissociation constant (*K*_d_) was calculated by fitting the amplitude data to the quadratic binding equation (middle panel). The plot of *k*_obs_ values versus S100A4 concentration (right panel) was used to obtain the binding constant *K*_1_ and the isomerization rate constants (*k*_2_ and *k*_-2_) by fitting a hyperbola to the data points. (C) Schematic illustration of the measured kinetic models for the N-ERMAD (left) and the isolated F2 lobe (right). After the binding of S100A4 dimer (subunits are red and pink) to F2 lobe (gray) a rapid isomerization of the complex occurs resulting in the structural rearrangement of not only F2 lobe, but also F1 and F3 lobes (light gray and dark gray, respectively).

The tryptophan fluorescence change upon S100A4 binding allowed us to determine the kinetic mechanism of the interaction with stopped-flow experiments. In fast-kinetic measurements, complex formation between the N-ERMAD and S100A4 was followed in real time. Using the amplitude component of the exponential fits, the equilibrium dissociation constant (*K*_d_) could be calculated, which was comparable with the value determined by steady-state fluorescence titration (*K*_d_ = 3.9 ± 0.9 μM and 2.2 ± 0.2 μM, respectively) (Fig 3A). Interestingly, the observed rate constant dependence on S100A4 concentration did not show the expected linear increase, but rather a hyperbolic shape. This behavior indicates an induced fit binding scenario where the binding event is followed by a structural isomerization of the complex [40]. According to this model, the complex formation can be characterized by a weak dissociation constant (*K*_1_ = 199 ± 21 μM), though it is followed by a highly favored isomerization step (*k*_2_ = 1.02 ± 0.04 s^-1^ and *k*_-2_ = 0.046 ± 0.007 s^-1^). Next, we measured the binding of S100A4 to the isolated F2 lobe (Fig 3B). Fitting the amplitude versus concentration plot to a quadratic binding equation yielded a *K*_d_ of 0.17 ± 0.07 μM. Again, *k*_obs_ was a hyperbolic function of the S100A4 concentration. However, both the binding and the isomerization steps were more favorable than in the case of the N-ERMAD (*K*_1_ = 9.9 ± 1.7 μM; *k*_2_ = 99 ± 5 s^-1^ and *k*_-2_ = 1.1 ± 2.0 s^-1^). Based on these findings, we can conclude that S100A4 binds to both the isolated F2 lobe and the N-ERMAD by an induced fit mechanism, but in the latter case the presence of F1 and F3 lobes put steric constraints on F2 subdomain conformation resulting in hampered binding of S100A4 and isomerization of the complex (Fig 3C).

### S100A4 also binds to the ezrin C-ERMAD

**Fig 4:**
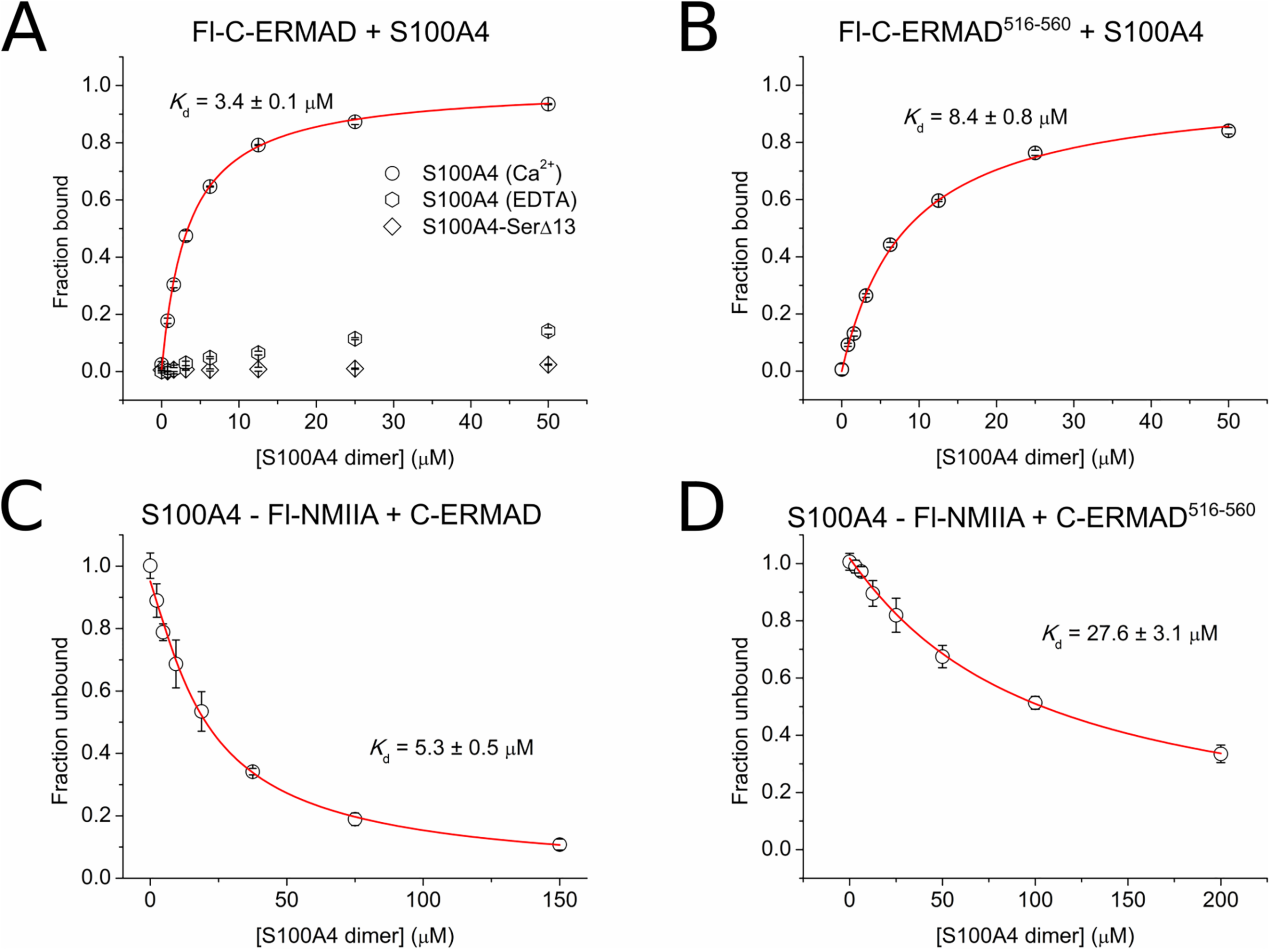
Interaction of the C-ERMAD with S100A4. (A) Fl-C-ERMAD (50 nM) was titrated with S100A4 and S100A4-SerΔ13. (B) Fl-C-ERMAD^516–560^ (50 nM) was titrated with S100A4. (C, D) Competitive FP measurement of Fl-NMIIA-bound S100A4 with the C-ERMAD and the C-ERMAD^516–560^, respectively. Each data point represents the mean ± SEM of three independent experiments. The data were fitted using a competitive binding equation (red line).

In order to characterize the potential interaction of the C-ERMAD with S100 proteins we carried out FP measurements using a fluorescently-labeled C-ERMAD construct (Lys516-Leu586, called Fl-C-ERMAD) as a tracer. It was found that S100A4 binds to Fl-C-ERMAD with micromolar affinity (*K*_d_ = 3.4 ± 0.1 μM) (Fig 4A). Similarly to the observations with N-ERMAD, no interaction was detected in the absence of Ca^2+^ or with S100A4-SerΔ13. S100P binds to Fl-C-ERMAD an order of magnitude weaker, while we could not detect any interaction with other S100 proteins (S2C Fig). Note that the binding of the N-ERMAD to Fl-C-ERMAD is in the nanomolar range (*K*_d_ = 8.6 ± 0.7 nM) (S2D Fig). To investigate whether the S100A4 binding site on the C-ERMAD overlaps with the actin binding site, we performed interaction studies with a shorter C-ERMAD fragment (C-ERMAD^516–560^), which lacks the actin-binding region [7]. Though S100A4 also interacts with this segment, the affinity was reduced by 2.5-fold (*K*_d_ = 8.4 ± 0.8 μM), which indicates that the S100A4 and actin binding sites partially overlap on the C-ERMAD (Fig 4B). Fl-C-ERMAD^516–560^ was also titrated with the N-ERMAD, however, no binding was detected (S2E Fig). To evaluate the potential effect of the fluorescent moiety conjugated to the C-ERMAD on its binding affinity, we carried out competitive FP measurements using Fl-NMIIA as a tracer and unlabeled C-ERMAD as a competitor (Fig 4C and 4D). Similarly to the results of direct FP measurements, competitive titrations revealed that the C-ERMAD truncation significantly affected S100A4 binding. However, the resulting dissociation constants were somewhat increased indicating that the fluorescent moiety enhanced the binding of Fl-C-ERMAD to S100A4 (*K*_d_ = 5.3 ± 0.3 μM and 28.6 ± 3.1 for the C-ERMAD and the C-ERMAD^516–560^, respectively).

Since both the fluorescent labeling at position 516 and truncation at position 560 affected the binding affinity of the C-ERMAD, we can conclude that an extended region of the C-ERMAD binds to S100A4 likely forming an asymmetric complex, similar to the N-ERMAD and to other recently characterized S100 protein-protein interactions [18, 41, 42]. Finally, we carried out binding experiments with Fl-C-ERMAD^T567D^ variant to evaluate the potential effect of Thr567 phosphorylation on S100A4 binding. Strikingly, we found that neither S100A4 nor the N-ERMAD showed hampered binding to this phosphomimetic C-ERMAD mutant (S2F and S2G Fig). The dissociation constants of ezrin domains in complex with wild-type S100A4 and other S100 proteins (including S100A4 variants) are summarized in Table 1 and S2J Fig, respectively.

**Table 1:** Interaction of wild-type S100A4 with ezrin constructs*.

	Trp fluorescence	FP	Competitive FP
N-ERMAD	2.2 ± 0.2 (3.9 ± 0.2)	–	1.1 ± 0.2
F2 lobe	< 0.1 (0.17 ± 0.07)	–	0.12 ± 0.08
C-ERMAD	–	3.4 ± 0.1	5.3 ± 0.5
C-ERMAD^516–560^	–	8.4 ± 0.8	27.6 ± 3.1
C-ERMAD^T567D^	–	2.7 ± 0.1	–
Ezrin	174.6 ± 23.3	–	92.1 ± 6.9
Ezrin^T567D^	157.6 ± 38.6	–	72.2 ±5.0

*Values represent the equilibrium dissociation constants (*K*_d_, mean ± SEM) in μM. The *K*_d_ values determined in stopped-flow experiment are shown in parentheses. For direct FP measurements, fluorescently-labeled C-ERMAD derivatives were used. In the competitive FP assays Fl-NMIIA^1908-1937^ was applied as a tracer.

### Conformational rearrangement in ezrin upon S100A4 binding

**Fig 5:**
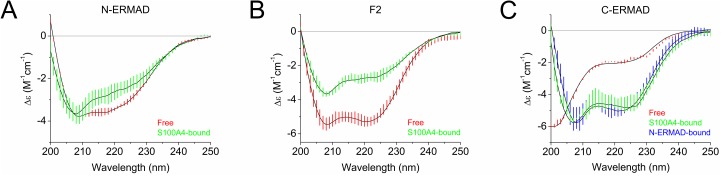
Structural changes in ezrin constructs upon S100A4 binding. (A) CD spectrum of free (red) and S100A4-bound (green) N-ERMAD. (B) CD spectrum of free (red) and S100A4-bound (green) F2 lobe. (C) CD spectrum of free (red), S100A4-bound (green) and the N-ERMAD-bound (blue) C-ERMAD. The CD spectra of the S100A4-bound ezrin fragments and the N-ERMAD-bound C-ERMAD were calculated by the subtraction of the CD spectrum of free S100A4 or N-ERMAD, respectively, from that of the complex assuming that the secondary structures of S100A4 and the N-ERMAD do not change upon complex formation. The colored vertical lines show the ± SEM of two independent experiments. The experimental data were fitted by the BeStSel software (black line).

Tryptophan fluorescence measurements indicated that S100A4 binding induces structural changes in the N-ERMAD. In order to characterize these alterations, CD spectroscopic measurements were performed and the CD spectra of the different ezrin constructs were analyzed using the BeStSel software. Deconvolution of the CD spectrum of the N-ERMAD resulted in an α-helix and β-sheet content of 25% and 17%, respectively. These values are lower than those calculated from the crystal structure of the N-ERMAD (36% and 22%, respectively; PDB ID: 4RMA) indicating that the solution structure of the N-ERMAD is less rigid than previously thought based on the crystallographic data. Upon addition of S100A4, the intensity of the CD signal at 220 nm was markedly lowered suggesting a decrease in the secondary structure content (SSC) of N-ERMAD (Fig 5A), although only subtle changes were calculated by BeStSel analysis. Similarly, the calculated α-helix content of the F2 lobe is 59% in the crystal structure, while in solution this value of the isolated F2 was measured to be 43% (Fig 5B, Table 2). Strikingly, upon S100A4 addition, a large drop in the SSC of F2 lobe occurred resulting in an α-helix content of 25%. These findings are in line with Trp fluorescence studies, where S100A4-binding induced a decrease in Trp fluorescence intensity indicating an increased solvent accessibility of Trp residues, i.e. the “melting” of the N-ERMAD and F2 lobe structures. We also investigated the effect of S100A4 binding on the SSC of the C-ERMAD. As it was expected based on the structure of other S100 protein interactions with disordered linear motifs [18, 42–45], the α-helix content of the C-ERMAD increased upon S100A4 binding from 10% to 37% (Fig 5C). For comparison, the α-helical content of the N-ERMAD-bound C-ERMAD was similarly high (50%). However, it was again lower than in the crystal structure of the full-length human ezrin (73%, PDB ID: 4RM8) (Fig 5C).

**Table 2:** Secondary structure contents of ezrin domains and S100A4 measured by CD spectroscopy*.

	α-helix (%)	β-sheet (%)	Turn (%)	Others (%)
N-ERMAD (15 μM)	24.6 (36)	16.5 (22)	15.9	42.9
N-ERMAD + S100A4 (50 μM dimer)	24.1	18.8	12.7	44.5
F2 lobe (25 μM)	43 (59)	3.9 (0)	12.2	40.9
F2 lobe + S100A4 (25 μM dimer)	24.7	7.5	14.9	52.9
C-ERMAD (25 μM)	9.9	0	17	73.1
C-ERMAD + N-ERMAD (30 μM)	49.9 (73)	0 (0)	13.5	36.6
C-ERMAD + S100A4 (100 μM dimer)	36.7	0	12.7	50.5

*Secondary structure contents were estimated using the BeStSel software. α-helix and β-sheet contents calculated from the crystal structures of human ezrin (PDB ID: 4RMA and 4RM8) are shown in parentheses.

### Structural analysis of the S100A4-ezrin complexes by NMR spectroscopy

**Fig 6:**
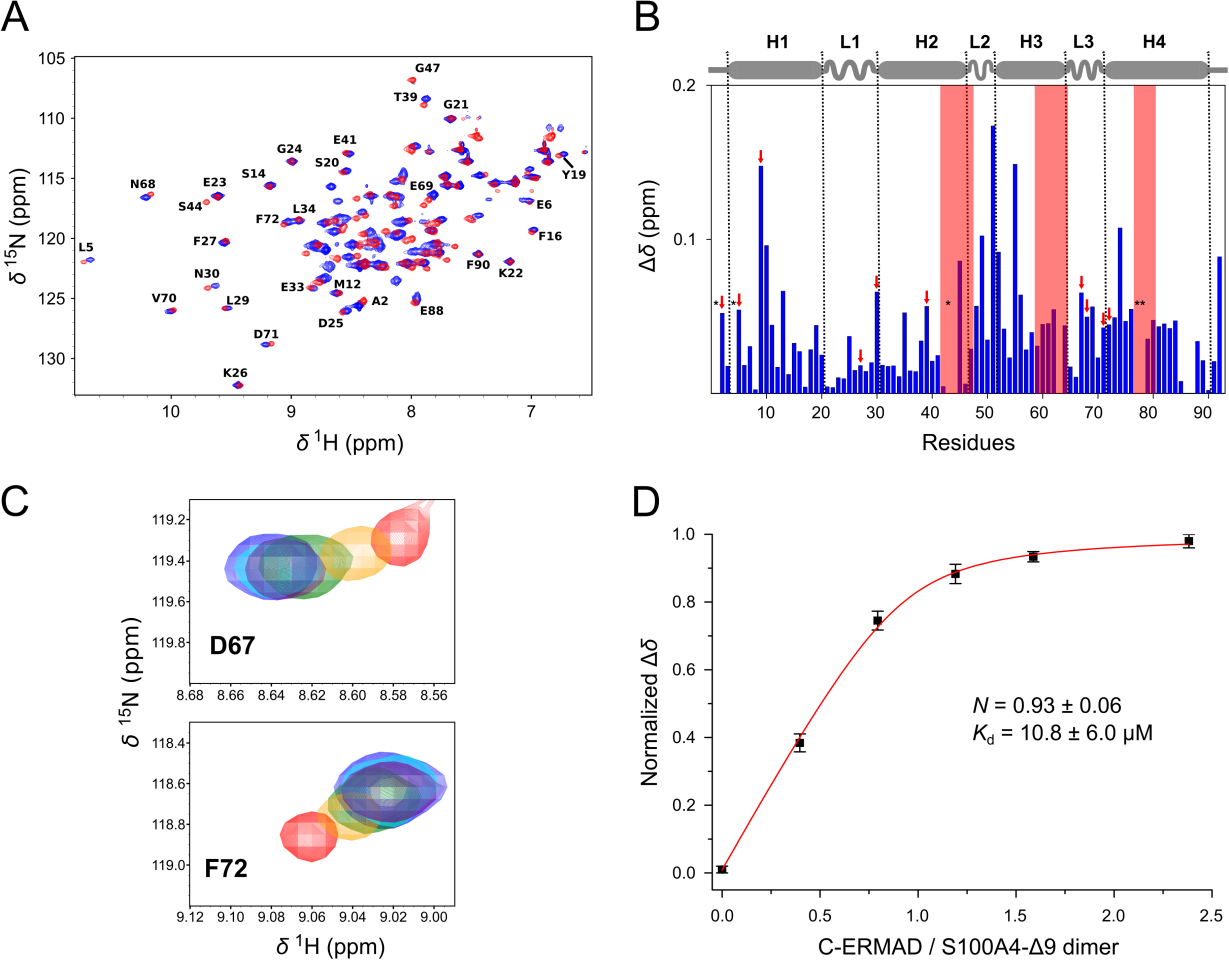
NMR spectroscopic analysis of the S100A4-C-ERMAD complex. (A) ^1^H-^15^N HSQC spectra of 0.4 mM free S100A4-Δ9 dimer (red) and upon addition of 0.6 mM C-ERMAD (blue) at 700 MHz and 300 K (for clarity not all assignments are shown) (B) Chemical shift mapping (Δ*δ*) of S100A4 peaks upon C-ERMAD binding. Red frames indicate the three regions that are significantly broadened upon complex formation (i.e. the half width of the Lorentzian peak in ^1^H dimension increased by more than 50%, for residues Ser44, Asp63, Val77 and Phe78). Asterisks show peaks that are either not detected or assignment is ambiguous. Secondary structural elements are shown above the graph. Red arrows show the residues of which the titration data were applied for the determination of binding parameters. (C) Titration of ^15^N-labeled S100A4-Δ9 with unlabeled C-ERMAD resulted in the gradual shift of peaks e. g. Asp67 and Phe72. The C-ERMAD / S100A4-Δ9 dimer ratio was 0 (red), 0.35 (orange), 0.7 (green), 1.05 (purple), 1.4 (cyan) and 2.1 (blue). (D) The mean ± SEM of the normalized chemical shift perturbations of ten S100A4-Δ9 residues (Ala2, Leu5, Leu9, Phe27, Asn30, Thr39, Asp67, Asn68, Asp71, Phe72) were plotted against the molar ratio of C-ERMAD to S100A4-Δ9 dimer. Red line indicates the fit to the quadratic binding equation yielding the binding affinity (*K*_d_) and stoichiometry (*N*).

While CD spectroscopic studies were used to detect structural changes in ezrin domains upon S100A4-binding, NMR spectroscopy was applied to investigate the effect of the complex formation on the structure of S100A4. For these measurements an S100A4 variant C-terminally truncated by 9 residues (S100A4-Δ9) was used due to the aggregation tendencies of the wild-type protein [32]. The truncated version shows similar binding affinities to the N-ERMAD and the C-ERMAD (as shown in S2H and S2I Fig, respectively). First, C-ERMAD was gradually added to ^15^N-labeled S100A4-Δ9 dimer up to a molar ratio of 2.1:1. ^1^H-^15^N HSQC spectra of both the free S100A4-Δ9 and the C-ERMAD-S100A4-Δ9 complex were successfully assigned: 87 peaks of 92 could be detected (except two prolines and Met1, Cys86, Asn87 residues) (Fig 6A). As expected, the mostly affected residues upon C-ERMAD binding are located in the hydrophobic pocket (comprising H3, H4 helices and L2 loop) where S100 interacting proteins bind (Fig 6B). Interestingly, significant chemical shift changes occur in the H1 helix, which is located far from the canonical ligand binding site. Similar tendencies were previously observed in the S100A4-NMIIA complex [32, 39], however, the Δ*δ* values were more significant for the myosin peptide.

The positions of the cross-peaks for the NH environments in comparison to those obtained for the free S100A4-Δ9 varied in different ways: (a), almost no change in peak position was observed: e.g. Ser14 (in H1), Glu23, Gly24 (in L1), (b) a shift in the peak position was detected e.g. Leu9 (in H1), Asp67 (in L3, Fig 6C), Phe72 (in H4, Fig 6C), and (c) several peaks significantly broadened, e.g. Gly47 (in L2) and Ser44 (in H2). Note that the gradual shift of cross-peaks indicates a fast chemical exchange process, i.e. the interconversion between C-ERMAD-bound and free S100A4-Δ9 is rapid relative to the NMR time scale, indicating that the C-ERMAD-S100A4 interaction can be characterized with a high dissociation rate constant [46]. Furthermore, the titration experiment allowed us to determine both the affinity and the stoichiometry of the interaction. These results indicate that one C-ERMAD chain binds to the S100A4 dimer (*N* = 0.93 ± 0.06) with a *K*_d_ value of 10.8 ± 6.0 μM (Fig 6D).

Although the results of the binding studies and the NMR titration experiments showed that the C-ERMAD binds to S100A4 in an asymmetrical manner, we could not detect doubled cross-peaks corresponding to the two S100A4 chains, as it has been previously demonstrated for the tight binding complexes of S100A10-AHNAK [47] and S100A4-NMIIA [32, 39]. On the one hand, this finding can also be explained by the fast-exchange binding of the C-ERMAD to the S100A4 dimer, which results in a similar environment (same cross-peak) of the residues belonging to the two S100A4 subunits. On the other hand, the above mentioned asymmetric complexes have very high (nanomolar) affinity with slow off-rate constant, leading to doubled peaks in the ^1^H-^15^N HSQC spectra (i.e. different environments of the given residue in each subunit).

**Fig 7:**
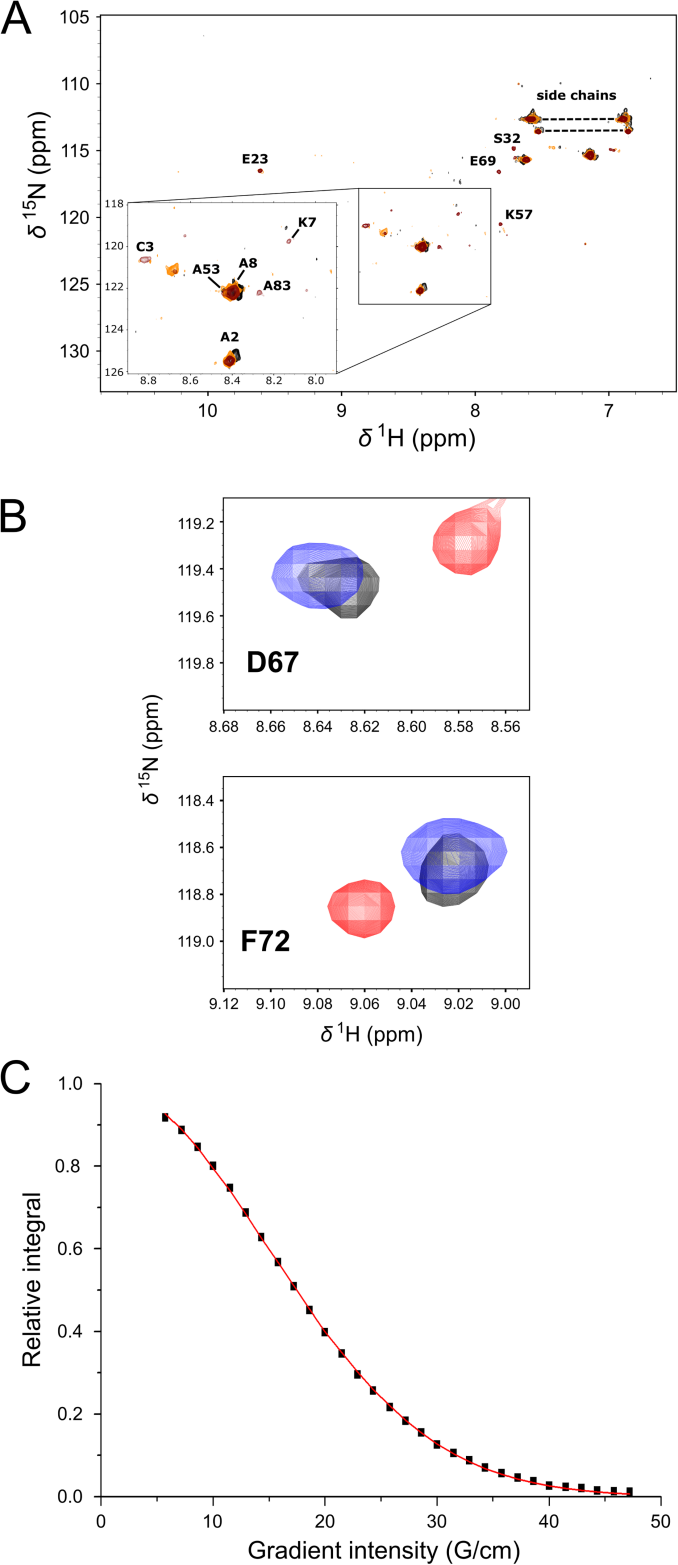
NMR spectroscopy analysis of the S100A4-N-ERMAD interaction. (A) ^1^H-^15^N HSQC spectra of the 1:1 complex of the N-ERMAD: S100A4-Δ9 dimer (black) and upon addition of C-ERMAD present in 1:1 (orange) and 1:2 molar ratio (maroon). Note that most peaks are broadened below the detection limit. (B) However, successive addition of the C-ERMAD results in the appearance of peaks that coincide to the positions in the C-ERMAD-S100A4-Δ9 complex: Asp67 and Phe72. Free S100A4-Δ9 peaks are shown in red, peaks of the C-ERMAD-S100A4-Δ9 complex are blue, while the peaks of S100A4-Δ9 in the presence of both the N-ERMAD and the C-ERMAD in a molar ratio of 1:1 and 1:2, respectively, are black. (C) A typical translational diffusion experiment representation for the C-ERMAD- S100A4-Δ9 complex integrated in the 1.892–1.276 ppm region: the decay of integrated signal intensity-gradient strength where squares represent measured points and the fitted Stejskal-Tanner equation (that leads to the determination of diffusion constant) is presented in a continuous red curve.

Subsequently, the formation of the N-ERMAD-S100A4-Δ9 complex was investigated. Due to the large size of the complex, leading to a significant line broadening, only a few peaks appear in the ^1^H-^15^N HSQC spectrum of S100A4-Δ9 (Fig 7A). Note that to overcome the peak broadening caused by N-ERMAD binding we attempted to use the F2 lobe for NMR studies, however, the S100A4-F2 lobe complex was precipitated at 100 μM concentrations. Successive addition of C-ERMAD to the N-ERMAD-S100A4-Δ9 complex resulted in the gradual appearance of certain peaks, many appearing at the same chemical shift values as those of the C-ERMAD-S100A4 complex, e.g. Ala8, Val13 in H1, Lys48 in L2, Ala53 in H3, Asp67 (Fig 7B) in L3, Phe72 (Fig 7B), Gln73, Ala83 in H4. This observation leads to the assumption that no ternary complex is formed, instead a mixture of binary complexes is present in the solution.

To further investigate the interaction of S100A4 with ezrin domains, we carried out diffusion measurements by NMR. The measured diffusion constant values (*D*) depend on the molecular weight of the species, the bigger the molecule, the lower the value of *D*. The calculated diffusion constants decreased in the order of S100A4-Δ9 dimer (22 kDa), the C-ERMAD-S100A4 complex (30 kDa) and the N-ERMAD-S100A4 complex (58 kDa) as expected, with values of (9.6 ± 0.3)· × 10^−11^ m^2^/s, (8.8 ± 0.3) × 10^−11^ m^2^/s and (6.5 ± 0.3) × 10^−11^ m^2^/s, respectively (a typical diffusion experiment with the fitting curve is shown in Fig 7C). Upon addition of the C-ERMAD to the N-ERMAD-S100A4 complex, the apparent diffusion constant increased to (7.9 ± 0.5) × 10^−11^ m^2^/s indicating again that S100A4 did not form ternary complex with the N-ERMAD and the C-ERMAD.

### S100A4 and full-length ezrin interact in cells

**Fig 8:**
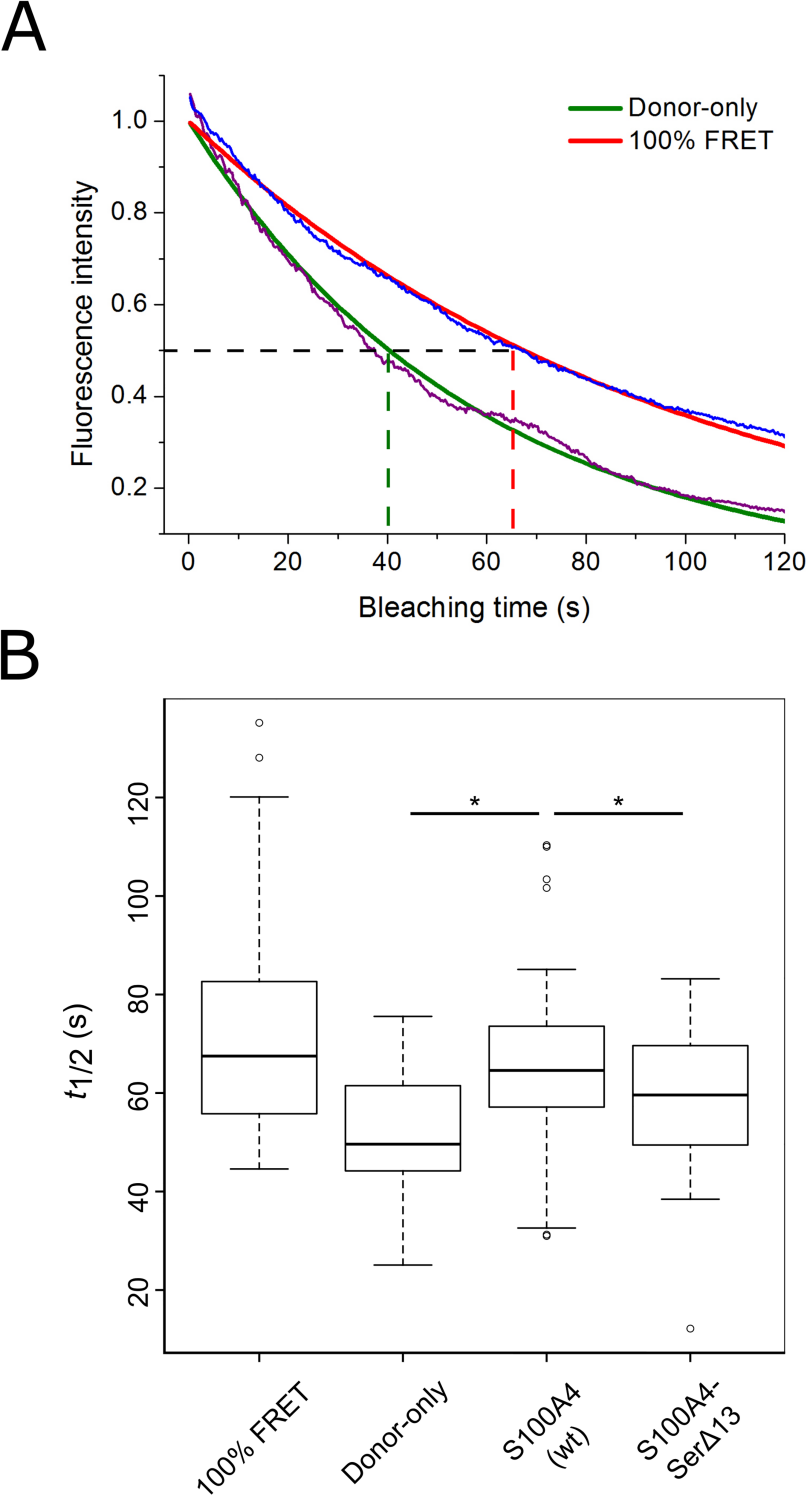
S100A4 interacts with ezrin in living HEK-293T cells. For FRET measurements, HEK-293T cells were co-transfected with pEGFP-ezrin and pmCherry-S100A4/S100A4-SerΔ13. As controls, cells transfected with pEGFP-ezrin alone (donor-only) or pEGFP-mCherry (100% FRET) were used. Donor photobleaching was performed by confocal microscopy, time constants of at least 80 ROI per sample were determined. (A) Two representative photobleaching curves from donor-only and 100% FRET samples (shown in violet and blue, respectively), the fitted exponential decay curves (green and red lines, respectively) and the calculated time constants (*t*_1/2_, marked with dashed lines). (B) Box-and-Whisker plots showing time constants of each sample were generated. Statistical analysis was performed using the Games-Howell test regarding the unequal sample size and inhomogeneous variance. Significant differences (p < 0.05) comparing S100A4 (wt) sample with others are marked with asterisk.

To confirm the interaction of S100A4 with ezrin in living cells, FRET (Förster resonance energy transfer) was measured in HEK-293T cells. This cell line does not overexpress either ezrin or S100A4, thus we co-transfected these cells with EGFP-ezrin along with mCherry-S100A4 or mCherry-S100A4-SerΔ13. A plasmid that encodes EGFP-mCherry fusion (with a linker of 7 amino acids) was also designed, and served as a positive control (pEGFP-mCherry, 100% FRET). Since several difficulties can be encountered using fluorescence intensity-based FRET measurements (e.g., unwanted spectral overlaps, extensive controls and calculations), the technique known as donor photobleaching was used [48]. In general, upon photobleaching, fluorescence intensity decreases; however, when FRET between the donor and acceptor fluorophores occurs, the donor molecule has an additional relaxation pathway, resulting in less pronounced photobleaching. Two representative curves of the fluorescence intensity during photobleaching indicating the difference in time constants are presented in Fig 8A. The quantification is based on determination of the bleaching time constants in cells that express only the donor fusion protein (EGFP-ezrin) and in cells expressing both fusions (EGFP-ezrin and mCherry-S100A4). The results show that cells expressing both ezrin and wild-type S100A4 show significantly higher *t*_1/2_ values as compared to cells expressing only ezrin (donor-only sample) or ezrin along with S100A4-SerΔ13 (Fig 8B), indicating that the two proteins indeed interact in HEK-293T cells.

To investigate the interaction of S100A4 with endogenously expressed ezrin, colocalization studies were performed in A431 epithelial carcinoma cells. To detect specific binding, S100A4 or the negative control (S100A4-SerΔ13) were transfected as an mCherry fusion (as described above). It was previously demonstrated that upon EGF (epidermal growth factor) stimulation, a shift in ezrin localization occurs from the cytosol into microvilli, membrane ruffles and adhesion sites [49]. Along this line, we stimulated serum-starved cells with EGF to visualize ezrin at discernible cellular structures. Indeed, upon activation, ezrin showed more distinct localization at adhesion sites near the cell membrane. As expected, wild-type S100A4 showed colocalization with stimulated ezrin, while the negative control non-binding S100A4-SerΔ13 did not show colocalization (S3 Fig). These results also confirm the specific binding of full-length ezrin and S100A4 in a cellular environment.

## Discussion

In the current work, we tested whether interactions occur between the ERM protein family member ezrin and five representatives (in addition to the previously characterized binding partner S100P) of the Ca^2+^-binding S100 family using full-length recombinant ezrin, as well as functional N- and C-terminal domains (N-ERMAD and C-ERMAD, respectively). We found that besides S100P, S100A4 also binds to the N-ERMAD of ezrin with micromolar affinity. Since there is a limited amount of data regarding S100 binding to a protein partner lacking any intrinsically disordered region [30, 50, 51], we used fast kinetic measurements to reveal, for the first time, the binding mechanism of an S100 protein to a folded protein partner. It was found that S100A4 binds to the N-ERMAD by an induced fit mechanism, where both the binding step to the effectively interacting F2 subdomain and the isomerization of the complex were hindered by the F1 and F3 subdomains. Regarding these data along with the results of CD spectroscopic measurements which showed a secondary structure content decrease in both the F2 lobe and the N-ERMAD upon S100A4-binding, we can conclude that not only the F2 lobe, but the whole N-ERMAD undergoes substantial structural changes presumably affecting the membrane binding properties of ezrin. This assumption is in accordance with the results of Austermann *et al*., who demonstrated that S100P competes with PIP_2_ for binding to ezrin [24]. Furthermore, Jia *et al*. found that the F2 subdomain comprises a selective S-nitrosylation site at Cys117 [25].This strictly conserved thiol side chain is solvent-inaccessible in the N-ERMAD structures [10, 52–54], therefore we hypothesize that the S-nitrosylation at Cys117 also requires the conformational rearrangement of the F2 lobe which can be mediated by S100A8/A9 binding. Nonetheless, for a detailed understanding of these structural changes the crystallization and 3D structure determination of the N-ERMAD-S100 complexes should be conducted.

Strikingly, we found that S100A4 binds not only to the N-ERMAD, but (unlike to S100P) to the C-ERMAD as well. The binding of S100A4 to the C-ERMAD appears to be a “regular” S100-partner interaction in a sense that a significant increase in the α-helix content of the intrinsically disordered C-ERMAD occurred upon complex formation. When the C-ERMAD was C-terminally truncated, i.e. the actin binding site was eliminated, the binding affinity of S100A4 to the C-ERMAD decreased 2.5–5.4-fold suggesting that S100A4-binding interferes with the ezrin-actin interaction. Investigation of the binding capability of S100A4 to the pseudo-phosphorylated C-ERMAD^T567D^ showed a similar binding strength as to the wild-type domain. This indicates that Thr567 phosphorylation does not modulate S100A4 binding. Moreover, the binding of N-ERMAD was also not altered, which finding is congruent with the hypothesis that membrane binding and phosphorylation of Thr567 act synergistically in ezrin activation [55].

Although the binding assays showed that S100A4 interacts asymmetrically with both functional domains of ezrin, one can assume that an S100A4 dimer is able to simultaneously bind the N-ERMAD and the C-ERMAD in the dormant state of ezrin freezing it in its inactive conformation. Using NMR spectroscopy, we clearly showed that S100A4 does not form ternary complex with the isolated domains of ezrin, therefore a single S100A4 dimer could not held the two ezrin domains together in an inactive conformation. Based on these results, we suggest a model in which two S100A4 dimers bind to the full-length ezrin: one to the N-ERMAD and the other to the C-ERMAD leading to the dissociation of the intramolecular N-ERMAD-C-ERMAD complex. However, we were able to detect only a very weak interaction between full-length ezrin and S100A4 in *in vitro* experiments. If we take into account that the affinity of the isolated N-ERMAD to the C-ERMAD is in the nanomolar range, and their intramolecular interaction is considerably tighter, this finding is reasonable even if S100A4 binds to both domains, however, only with micromolar affinities. Note that phosphomimicking the Thr567 phosphorylation did not affect the binding of S100A4 to C-ERMAD nor to the full length ezrin.

**Fig 9:**
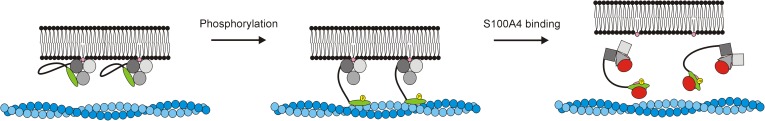
Proposed mechanism of ezrin regulation by S100A4-binding. In the dormant state, ezrin shows limited cross-linking activity of the actin cytoskeleton (blue) to the plasma membrane. PIP_2_ is bound by the F1 (light gray) and the F3 (dark gray) subdomains. The actin-binding site on the C-ERMAD is masked by the N-ERMAD lobes F2 (gray) and F3 (dark grey). Upon phosphorylation of Thr567 (yellow), ezrin opens up to acquire its active conformation. Binding of S100A4 to the N-ERMAD and the C-ERMAD leads to allosteric and direct inhibition of ezrin function, respectively.

Importantly, we demonstrated that ezrin and S100A4 interact in a complex cellular environment. To resolve the contradiction observed between the results of *in vitro* and *in vivo* experiments, we propose the following model for the S100A4-mediated regulation of ezrin function (Fig 9). The tight intramolecular interaction of the N-ERMAD and the C-ERMAD presumably holds ezrin in a kinetically blocked state, which interferes not only with S100A4-binding, but, as it was previously suggested, with actin-binding as well [55]. However, even in the dormant state, ezrin is recruited to PIP_2_-containing membranes and shows limited actin binding [55]. When phosphorylation events take place (e.g. phosphorylation of Thr567), ezrin tends to adopt an open conformation and binds to actin filaments cross-linking the cytoskeleton to the plasma membrane. Similarly to actin-binding, the interaction of S100A4 with ezrin might be more favorable in its membrane-bound and/or phosphorylated state. In this way, S100A4-binding could contribute to the Ca^2+^-dependent fine-tuning of this complicated activation mechanism by changing the structure of the N-ERMAD and at least partially masking the actin-binding site in the C-ERMAD, and thus hindering PIP_2_- and actin-binding events, respectively.

Since each S100 paralog has a cell-specific expression pattern [17], their potential coexistence in a certain cell type and high sequence similarity could imply both partially overlapping and partially distinct functions through their interactions with common protein partners. A notable example is the tumor suppressor p53 that interacts with numerous S100 paralogs. Here, S100 proteins bind to both the transactivation and tetramerization domains of p53 with distinct preference, moreover, the p53 homologs p63 and p73 also bind to S100 proteins with likely partially different and overlapping functions [56, 57]. Similarly, annexins and non-muscle myosin II paralogs also have a set of S100 protein partners showing paralog specificity from both sides [28, 58–60]. Our present findings seem to be in line with this view. Ezrin interacts with multiple S100 partners, however the N- and C-terminal domains differentiate of their S100 partners. The N-ERMAD binds to both S100A4 and S100P, while the C-ERMAD interacts only with S100A4 with likely similar (hindering PIP_2_-binding) as well as different functional consequences (activation vs. inhibition of actin-binding). It remains to be seen whether S100 proteins could interact with other ERM proteins showing high sequence homology to ezrin. Since several members of both the S100 and ERM protein families are implicated in cancer progression [61, 62], the characterization of these putative interactions could contribute to the better understanding of not only the role of S100 proteins in the regulation of cytoskeletal dynamics together with ERM proteins, but also the molecular events leading to cancer and metastasis.

## Supporting information

S1 Fig SDS-PAGE analysis of the recombinant proteins used in this study. 5 μg of ezrin (A) or S100 proteins (B) were loaded onto a 10% Tris-Tricine gel. (A) 1: ezrin (full-length), 2: ezrin^T567D^, 3: N-ERMAD, 4: F2, 5: C-ERMAD, 6: C-ERMAD^T567D^, 7: C-ERMAD^516–560^. (B) 1: S100A2, 2: S100A4, 3: S100A4-Δ9, 4: S100A4-SerΔ13, 5: S100A6, 6: S100B, 7: S100P. Note that in the case of C-ERMAD fragments and certain S100 samples, a band corresponding to the oxidized dimer is also detectable.
(TIF)

S2 Fig Interaction assays of S100 proteins with ezrin domains. (A) 2 μM N-ERMAD was titrated with various S100 proteins and a decrease in the intrinsic tryptophan fluorescence intensity was detected. (B) Fl-NMIIA (50 nM) peptide was titrated with wild-type S100A4 and an increase in fluorescence polarization (FP) signal was monitored. (C, D) Fl-C-ERMAD (50 nM) was titrated with various S100 proteins or N-ERMAD, respectively, in FP assays. (E) Fl-C-ERMAD^516–560^ (50 nM) was titrated with N-ERMAD in FP assay. (F, G) Fl-C-ERMAD^T567D^ (50 nM) was titrated with N-ERMAD and S100A4, respectively, and the FP signal was detected. (H) N-ERMAD (2 μM) was titrated with S100A4-Δ9 in a steady-state tryptophan fluorescence measurement. (I) Binding of Fl-C-ERMAD (50 nM) to S100A4-Δ9 was determined in FP assay. (J) Summary of the *K*_d_ (μM) values of the interactions between ezrin domains and S100 paralogs or mutants. Each data point represents the mean ± SEM of three independent experiments. *K*_d_ values were calculated by fitting the data to a quadratic binding equation using software Origin Pro8 (OriginLab Corp.).
(TIF)

S3 Fig Colocalization study of endogenously expressed ezrin and S100A4 in A431 epithelial carcinoma cells. A431 cells were transfected with pmCherry-S100A4 or pmCherry-S100A4-SerΔ13 (red). After 24 h, serum-starved cells were stimulated with EGF. Fixed cells were immunostained with anti-ezrin antibody and Alexa-488 conjugated secondary antibody (green). Nuclei were stained by DAPI (blue). Images were taken by Zeiss AxioImager Z1 microscope. Arrows show ezrin localization at cell adhesion sites. Scale bar represents 10 μm.
(TIF)
