# Supplementary figures and images for: Ezrin interacts with S100A4 via both its N- and C-terminal domains
Source: PLoS One. 2017 May 11;12(5):e0177489. doi: 10.1371/journal.pone.0177489 (PMC5426754; doi:10.1371/journal.pone.0177489)

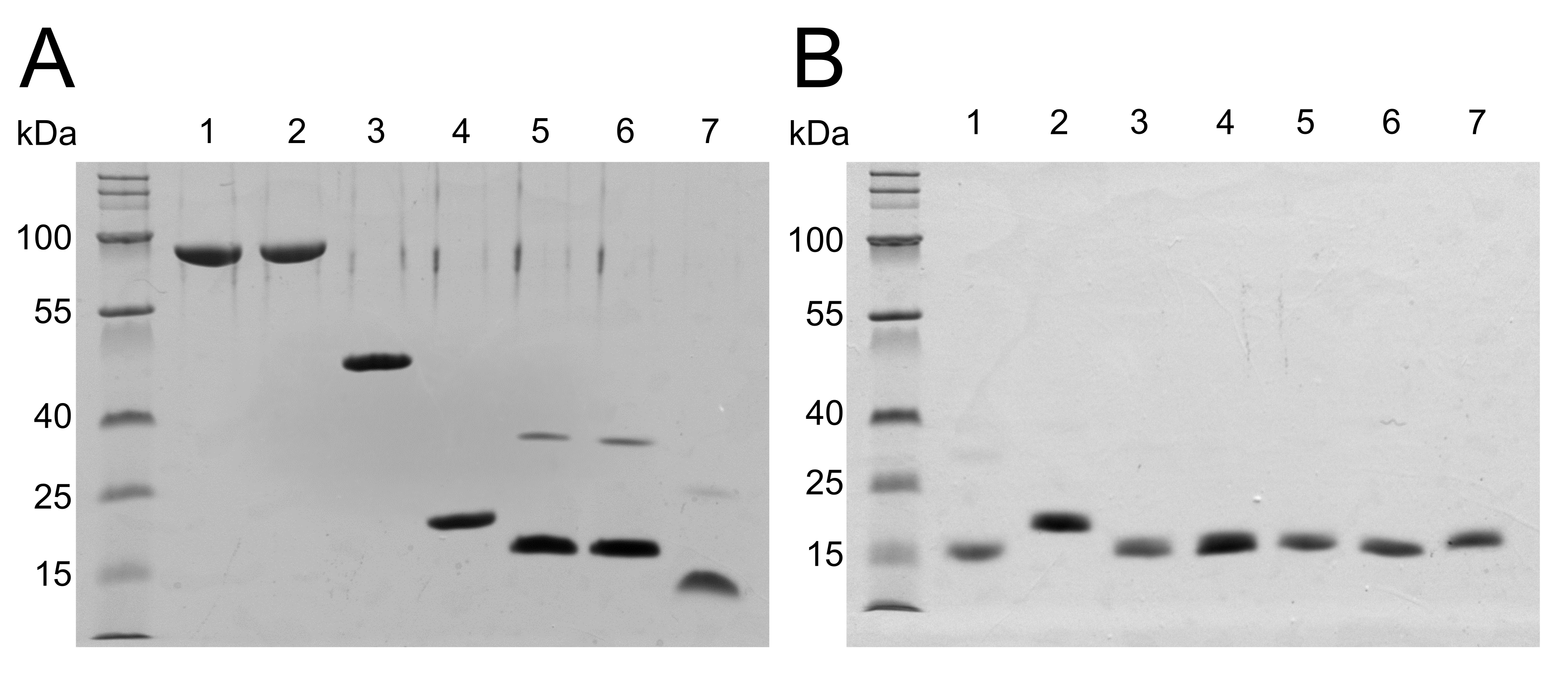

Supplement: S1 Fig — 5 μg of ezrin (A) or S100 proteins (B) were loaded onto a 10% Tris-Tricine gel. (A) 1: ezrin (full-length), 2: ezrinT567D, 3: N-ERMAD, 4: F2, 5: C-ERMAD, 6: C-ERMADT567D, 7: C-ERMAD516–560. (B) 1: S100A2, 2: S100A4, 3: S100A4-Δ9, 4: S100A4-SerΔ13, 5: S100A6, 6: S100B, 7: S100P. Note that in the case of C-ERMAD fragments and certain S100 samples, a band corresponding to the oxidized dimer is also detectable. (TIF) [file pone.0177489.s001.tif]

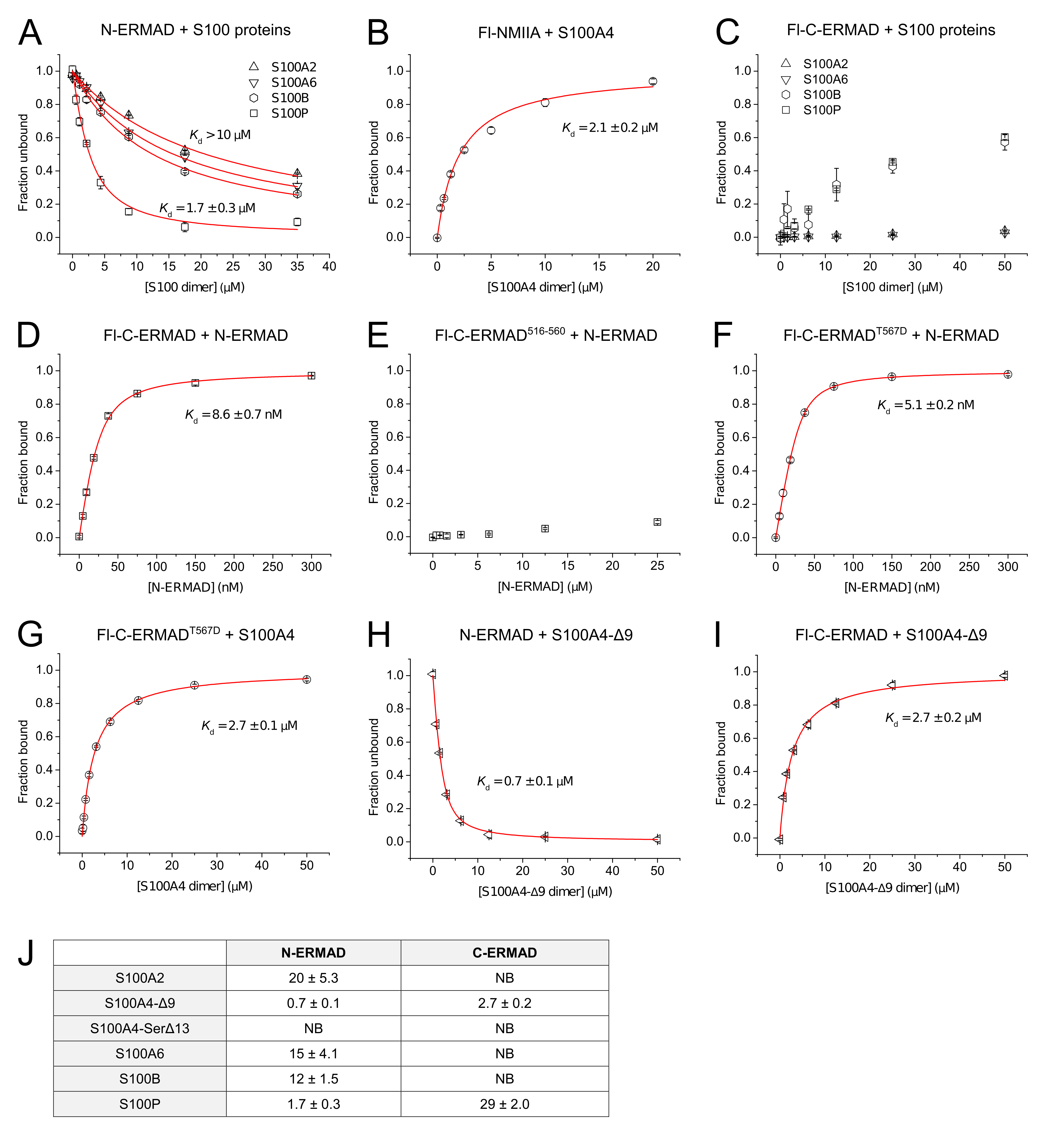

Supplement: S2 Fig — (A) 2 μM N-ERMAD was titrated with various S100 proteins and a decrease in the intrinsic tryptophan fluorescence intensity was detected. (B) Fl-NMIIA (50 nM) peptide was titrated with wild-type S100A4 and an increase in fluorescence polarization (FP) signal was monitored. (C, D) Fl-C-ERMAD (50 nM) was titrated with various S100 proteins or N-ERMAD, respectively, in FP assays. (E) Fl-C-ERMAD516–560 (50 nM) was titrated with N-ERMAD in FP assay. (F, G) Fl-C-ERMADT567D (50 nM) was titrated with N-ERMAD and S100A4, respectively, and the FP signal was detected. (H) N-ERMAD (2 μM) was titrated with S100A4-Δ9 in a steady-state tryptophan fluorescence measurement. (I) Binding of Fl-C-ERMAD (50 nM) to S100A4-Δ9 was determined in FP assay. (J) Summary of the Kd (μM) values of the interactions between ezrin domains and S100 paralogs or mutants. Each data point represents the mean ± SEM of three independent experiments. Kd values were calculated by fitting the data to a quadratic binding equation using software Origin Pro8 (OriginLab Corp.). (TIF) [file pone.0177489.s002.tif]

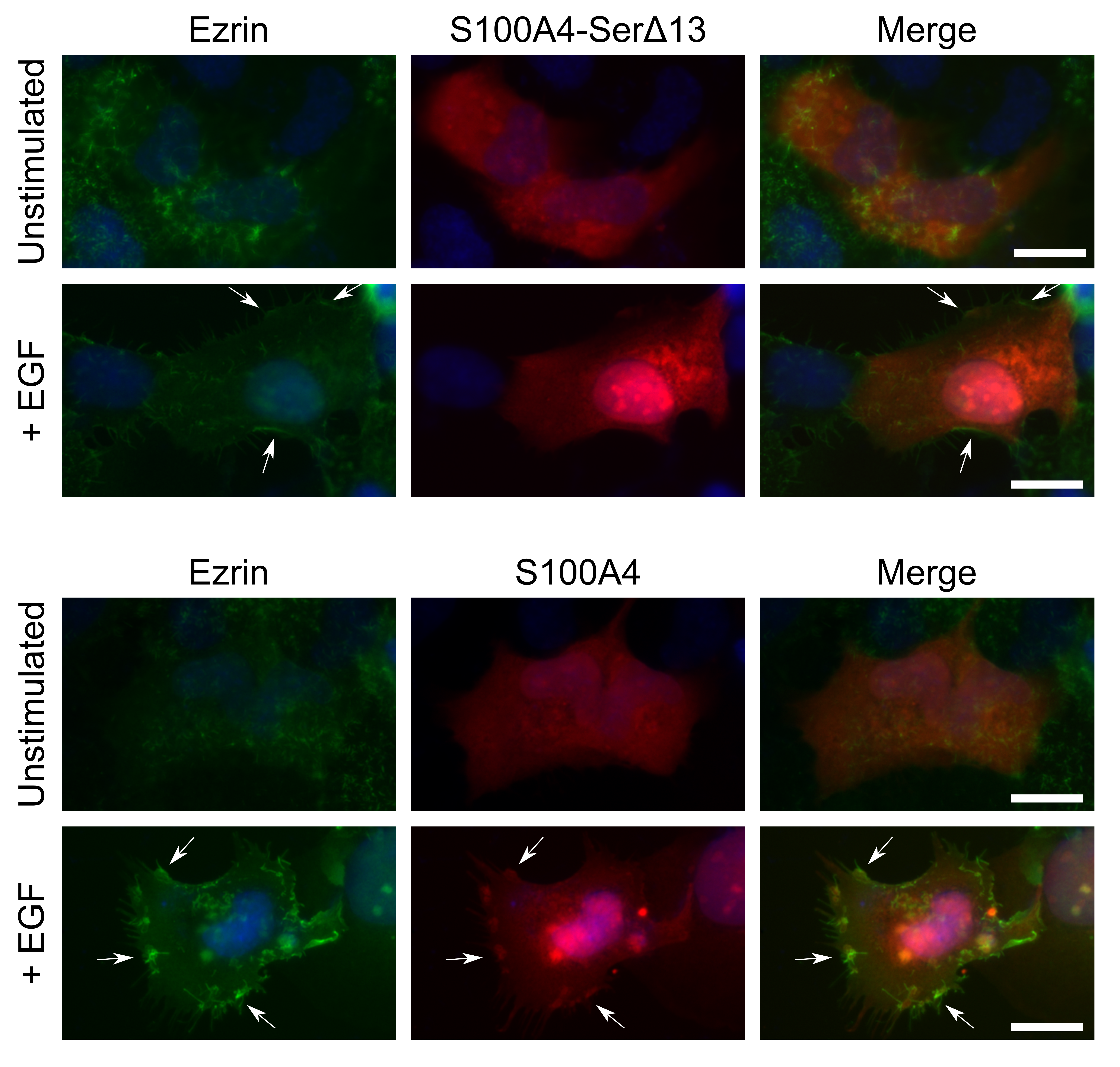

Supplement: S3 Fig — A431 cells were transfected with pmCherry-S100A4 or pmCherry-S100A4-SerΔ13 (red). After 24 h, serum-starved cells were stimulated with EGF. Fixed cells were immunostained with anti-ezrin antibody and Alexa-488 conjugated secondary antibody (green). Nuclei were stained by DAPI (blue). Images were taken by Zeiss AxioImager Z1 microscope. Arrows show ezrin localization at cell adhesion sites. Scale bar represents 10 μm. (TIF) [file pone.0177489.s003.tif]
